# Alphalipoic Acid Prevents Oxidative Stress and Peripheral Neuropathy in Nab-Paclitaxel-Treated Rats through the Nrf2 Signalling Pathway

**DOI:** 10.1155/2019/3142732

**Published:** 2019-02-10

**Authors:** Hong Sun, Xi Guo, Ziteng Wang, Peipei Wang, Zhe Zhang, Jihong Dong, Rongyuan Zhuang, Yuhong Zhou, Guo Ma, Weimin Cai

**Affiliations:** ^1^Department of Clinical Pharmacy, School of Pharmacy, Fudan University, Shanghai 201203, China; ^2^Department of Medical Oncology, Zhongshan Hospital, Fudan University, Shanghai 200032, China; ^3^Department of Neurology, Zhongshan Hospital, Fudan University, Shanghai 200032, China

## Abstract

Peripheral neuropathy is the major dose-limiting side effect of paclitaxel (PTX), affecting both the quality of life and the survival of cancer patients. Nab-paclitaxel (nab-PTX) was developed to provide additional clinical benefits and overcome the safety drawbacks of solvent-based PTX. However, the prevalence of peripheral neuropathy induced by nab-PTX was reported higher than that induced by solvent-based PTX. Upon investigation, oxidative stress plays a major role in the toxicity of nab-PTX. In order to assess if the antioxidant alphalipoic acid (*α*-LA) could prevent the nab-PTX-induced peripheral neuropathy, Sprague-Dawley (SD) rats were treated with three doses of *α*-LA (15, 30, and 60 mg/kg in normal saline, i.p., q.d. (days 1-30)) and/or nab-PTX (7.4 mg/kg in normal saline, i.v., q.w. (days 8, 15, and 22)). Body weight and peripheral neuropathy were measured and assessed regularly during the study. The assessment of peripheral neuropathy was performed by the von Frey and acetone tests. A tumor xenograft model of pancreatic cancer was used to assess the impact of *α*-LA on the antitumor effect of nab-PTX. Results showed that *α*-LA significantly ameliorated the peripheral neuropathy induced by nab-PTX (*p* < 0.05) without promoting tumor growth or reducing the chemotherapeutic effect of nab-PTX in a tumor xenograft model. Moreover, *α*-LA might significantly reverse the superoxide dismutase (SOD), glutathione (GSH), and malondialdehyde (MDA) levels altered by nab-PTX in the serum and the spinal cord of rats. Furthermore, *α*-LA could reverse the mRNA and protein expressions of Nrf2 (nuclear factor erythroid 2-related factor 2) and three Nrf2-responsive genes (HO-1, *γ*-GCLC, and NQO1) altered by nab-PTX in the dorsal root ganglion (DRG) of rats. In conclusion, our study suggests that *α*-LA could prevent oxidative stress and peripheral neuropathy in nab-PTX-treated rats through the Nrf2 signalling pathway without diminishing chemotherapeutic effect.

## 1. Introduction

Paclitaxel (PTX) is classified as a microtubule-binding agent, which is widely used to treat several solid tumors including breast, ovarian, and lung cancers [1–3]. Its primary antitumor effect occurs by disrupting the mitotic spindle and microtubule dynamics, leading to apoptosis [4–6]. Peripheral neuropathy, a painful and major dose-limiting side effect of PTX treatment, is predominantly sensory and worsens with cumulative dosing [7]. The typical symptoms of peripheral neuropathy include bilateral numbness, tingling, evoked pain, and spontaneous pain to mechanical and cold stimuli in the hands and/or feet [8, 9]. Peripheral neuropathy can persist for months or years following cessation of PTX. At present, no effective treatments exist to prevent the development of PTX-induced neuropathy or reverse it once established. Therefore, the emergence of peripheral neuropathy during PTX therapy often results in the discontinuation of otherwise successful chemotherapy, thus impacting both the quality of life and the survival of cancer patients [10–12].

It was reported that peripheral neuropathy was associated with both the solvent (Cremophor EL) and PTX itself [13]. As a modified formulation of PTX, nanoparticle albumin-bound paclitaxel (nab-PTX) is a water-soluble and Cremophor EL-free formulation of PTX which has no toxicities induced by Cremophor EL [14–16]. However, various studies, including our preliminary experiment, found that the prevalence and severity of peripheral neuropathy induced by nab-PTX were higher than those induced by solvent-based PTX [17, 18]. Hence, more attention should be given to peripheral neuropathy during nab-PTX treatment. Effective measures must be explored and discovered to prevent the development of peripheral neuropathy induced by nab-PTX.

The exact mechanism of peripheral neuropathy induced by paclitaxel has not been fully elucidated [19, 20]. In recent years, oxidative stress has been considered a significant factor responsible for chemotherapy-induced peripheral neuropathy [21–24]. Some antioxidants, such as glutathione and *N*-acetylcysteine, have been used for the prevention and treatment of chemotherapy-induced peripheral neuropathy [25, 26]. As a “universal antioxidant,” alphalipoic acid (*α*-LA) diminishes the harmful effects of oxidative stress in diabetic neuropathy [27, 28]. Not only does it act on pain, but, as a pathogenesis-oriented treatment, it also improves other symptoms, like paraesthesiae and numbness, along with sensory deficits and muscle strength [27]. In addition, the nuclear factor erythroid 2-related factor 2 (Nrf2)/antioxidant response element signalling pathway is the main mechanism preventing the effects of oxidative stress. Deleting Nrf2 gene expression led to an increase in neural oxidative stress and rendered relatively less axonal regeneration [29]. Therefore, enhancing nuclear Nrf2 expression and subsequent oxidative stress inhibition may be an important approach in preventing oxidative neural damage and promoting the repair process after peripheral neuropathy. Furthermore, *α*-LA was reported to attenuate oxidative damage by activating the Nrf2/HO-1 pathway [30].

Due to the similar pathogenesis between diabetic neuropathy and PTX-induced peripheral neuropathy, *α*-LA might also have a therapeutic effect on PTX-induced peripheral neuropathy. Considering the severity of peripheral neuropathy induced by nab-PTX compared with solvent-based PTX, the objective of this study was to investigate whether *α*-LA as a neuroprotective agent and pretreatment can reduce the peripheral neuropathy induced by nab-PTX while determining the underlying molecular mechanisms of the compound's neuroprotection. Additionally, we sought to determine whether treatment with *α*-LA would have any effects on the chemotherapeutic effect of nab-PTX.

## 2. Materials and Methods

### 2.1. Drugs and Reagents

The alphalipoic acid injection (Yabao Pharmaceutical Group Co. Ltd.), nab-paclitaxel injection (Fresenius Kabi USA, LLC), and normal saline injection (Tianrui Pharmaceutical, Zhejiang, China) were obtained from Zhongshan Hospital of Fudan University (Shanghai, China). The mRNA extraction kit, cDNA extraction kit, RNA amplification kit, primer design, and synthesis were provided by Takara (Takara Bio Inc., Shiga, Japan). Protein antibodies were purchased from Abcam Inc. (Cambridge, MA, USA).

### 2.2. Experimental Design

Adult male Sprague-Dawley (SD) rats were purchased from Sippr-BK Experimental Animal Center (Shanghai, China) and raised under standard conditions of animal housing (a 12-hour light/dark cycle, temperature 25°C, and humidity 55–60%) with free access to food and water. All animal care and experimental protocols were conducted in accordance with the Institutional Animal Care and Use Committee (IACUC), School of Pharmacy, Fudan University. The ethical approval was shown in Supplementary File 1. The experimental procedures complied with the recommendations of the International Association for the Study of Pain [31]. All efforts were made to minimize the number of animals used and their suffering. After 1 week of circumstance adaption, SD rats were randomly assigned to 5 groups according to body weight: vehicle (normal saline), nab-PTX (7.4 mg/kg in normal saline, i.v., q.w (days 8, 15, and 22)), and nab-PTX (7.4 mg/kg in normal saline, i.v., q.w. (days 8, 15, and 22)) combined with three doses of *α*-LA (low dose,15 mg/kg; middle dose, 30 mg/kg; and high dose, 60 mg/kg, respectively, in normal saline, i.p., q.d. (days 1-30)) (Supplementary Figure 1). The body weight of rats was measured every 3 to 4 days. The assessment of peripheral neuropathy was performed blind with respect to the drug administration on days 8, 15, 22, and 29 (days 1, 7, 14, and 21 after nab-PTX administration). The animals were double-labelled, and then, the final data was back-analyzed with the original groups of the animals.

### 2.3. von Frey Test for Mechanical Allodynia

All rats were allowed to acclimate for approximately 30 minutes before testing. The mechanical paw withdrawal threshold was assessed using the von Frey filaments (U.S. North Coast, NC12775-99). Each rat was placed in a chamber (20 × 10 × 20 cm) with a customized platform made of iron wires, which create a 10 mm grid throughout the entire area. A series of 7 calibrated von Frey filaments were applied to the central region of the plantar surface of one hind paw in ascending order (1, 2, 4, 6, 8, 10, and 15 g) with the highest filament at 15 g. Each filament was applied to the midplanter skin of each hind paw five times until the force slightly bent the tip and was then held for 5 seconds. A trial consisted of applying a von Frey filament to the hind paw 5 times at 15 sec intervals. When the hind paw withdrew from a particular filament in 4 of the 5 consecutive applications, the value of that filament in grams was considered to be the paw withdrawal threshold (PWT). Withdrawal responses from both hind paws were counted, and the percentage response was calculated as previously described [32, 33]. The test was performed on days 1, 7, 14, and 21 after nab-PTX administration.

### 2.4. Acetone Test for Cold Hypersensitivity

The responses to an acetone droplet on the hind paw were measured for the assessment of cold hypersensitivity [34]. The use of acetone for cold hypersensitivity in the peripheral and central neuropathic pain animal models was examined and suggested to be similar to the response seen in human neuropathic pain patients who suffered from mechanical and cold hypersensitivity in previous studies [35, 36]. The rats were placed in individual areas and allowed to acclimate for at least 30 minutes. For testing, 50 *μ*l of acetone was applied to the plantar skin of the rat's hind paw slowly over a period of 2–3 seconds by a pipette. The injured rats quickly lifted and vigorously shook the paw after the application of the acetone to the hind paw. The uninjured rats generally showed no lifting response to the application. Both the left and right hind paws were tested with at least 5 minutes between each application of acetone. The frequency of responses was calculated from the number of times the rat responded to an acetone application to the hind paw skin out of 5 trials (response frequency (%) = (number of response/5 acetone trials) × 100). Only reflexive responses that also included head orientation, vocalization, or grooming of the tested limb were counted as a positive response.

### 2.5. Oxidative Activity Evaluation by Biochemical Assessment

As markers of oxidative stress, the superoxide dismutase (SOD), glutathione (GSH), and malondialdehyde (MDA) levels of the serum and the spinal cord of the SD rats were measured by a microplate reader (Thermo Fisher Scientific) using the assay kits (Nanjing Jiancheng Bioengineering Research Institute, Nanjing, China) according to the procedure recommended by the manufacturer (SOD, 450 nm; GSH, 405 nm; and MDA, 530 nm). The concentrations of SOD, GSH, and MDA were calculated in nanomoles per gram of protein.

### 2.6. Western Blotting

After mechanical and cold allodynia testing on day 21, the rats were deeply anesthetized with an i.p. injection of 10% chloral hydrate. For RNA assays, the bilateral DRGs (L4-6) were harvested, immediately frozen with liquid nitrogen, and then stored at -80°C. Proteins were extracted from DRGs by cell lysis buffer for Western blotting and IP (Dalian Meilun Biological Technology Co. Ltd, China) according to the manufacturer's protocol. Protein concentrations were measured using a bicinchoninic acid (BCA) protein assay kit (Beyotime, Jiangsu, China). Total protein was electrophoresed in a 10% SDS-PAGE gel and transferred onto polyvinylidene difluoride (PVDF) membranes (Millipore, US). The membranes were blocked with 5% nonfat milk in TBST. The membranes were incubated with specific primary antibodies overnight at 4°C. After the membranes were washed with PBST, they were incubated with HRP-conjugated secondary antibodies for 2 hours at room temperature and then washed 3 more times again. The antigen-antibody complexes were detected by enhanced chemiluminescence (ECL) (Amersham Life Science, England) and visualized by Bio-Rad ChemiDoc XRS (Bio-Rad, USA).

### 2.7. Quantitative Real-Time Polymerase Chain Reaction (qRT-PCR)

The total RNA of the bilateral dorsal root ganglions (DRGs) (L4-6) was isolated by RNAiso Plus (Takara Bio Inc., Shiga, Japan) according to the manufacturer's instruction. The first-strand cDNA was generated using the PrimerScript™ RT Reagent kit with gDNA Eraser (Takara Bio Inc., Shiga, Japan). The sequences of the primers used for Nrf2, HO-1, *γ*-GCLC, NQO1, and GAPDH were shown in Table 1. The Bio-Rad iCycler qPCR system and TB Green Premix EX Taq II (Takara Bio Inc., Shiga, Japan) were used to perform qPCR. The cycling conditions for all primer pairs were as follows: 5 seconds at 95°C and 30 seconds at 60°C, according to the manufacturer's protocol. The ratios of Nrf2, HO-1, *γ*-GCLC, and NQO1 mRNA expressions to the GAPDH level in each sample were considered to be the mRNA levels and were expressed relative to the mRNA levels of the control group. Data were shown as mean ± standard deviation.

### 2.8. Chemotherapeutic Effect Evaluation

After determining the neuroprotective effects of *α*-LA, we further investigated whether treatment with *α*-LA would have any effects on the chemotherapeutic effect of nab-PTX through the establishment of a subcutaneous xenograft model of pancreatic cancer.

The pancreatic cancer cell line CFPAC-1 was purchased from the Cell Bank of Shanghai Institute of Cell Biology, Chinese Academy of Sciences (Shanghai, China). CFPAC-1 cells were cultured in IMDM (Hyclone, China) with 10% (*v*/*v*) FBS and incubated at 37°C in 5% CO_2_ in a CO_2_ incubator (Thermo Scientific Forma). Cells were routinely checked for *Mycoplasma* contamination.

CFPAC-1 (5 × 10^6^ cells) was injected subcutaneously into the right of Sippr-BK Balb/c nude mice (6-7 weeks old). Cohorts of athymic mice with an average tumor volume of approximately 150-200 mm^3^ were randomized to 4 groups according to the tumor volume: vehicle (normal saline), *α*-lipoic acid (60 mg/kg in normal saline, i.p., q.d (days 1-30)), nab-paclitaxel (7.4 mg/kg in normal saline, i.v., q.w (days 8, 15, and 22)), and their combination (*α*-lipoic acid, 60 mg/kg in normal saline, i.p., q.d (days 1-30); nab-paclitaxel, 7.4 mg/kg in normal saline, i.v., q.w (days 8, 15, and 22)). The body weight and tumor volume of the mice were measured and recorded 2 to 3 times per week. The tumor volume measurement was performed blind with respect to the drug administration. The animals were double-labelled, and then, the final data was back-analyzed with the original groups of the animals. Tumor volume was calculated using the following formula: tumor volume = (length × width^2^)/2. At the end of the experiment, the mice were sacrificed and tumor xenografts were removed and weighed (Supplementary Figure 2 ).

### 2.9. Statistical Analysis

Experimental data were presented as mean ± standard deviation from at least 3 independent experiments. One-way ANOVA followed by S-N-K multiple comparisons was used to evaluate the statistical significance of the differences among multiple groups *in vitro*, and one-way ANOVA followed by Dunnett's multiple comparisons was applied to the *in vivo* comparisons among multiple groups. A *p* value less than 0.05 was considered to be statistically significant.

## 3. Results

### 3.1. Body Weight and Peripheral Neuropathy Assessment

The administration of alphalipoic acid, nab-paclitaxel, and vehicle was well tolerated by the rats, without any cases of mortality. Rats that received nab-paclitaxel showed a slight but insignificant reduction in body weight compared with that of the controls (Supplementary Figure 3).

**Figure 1:**
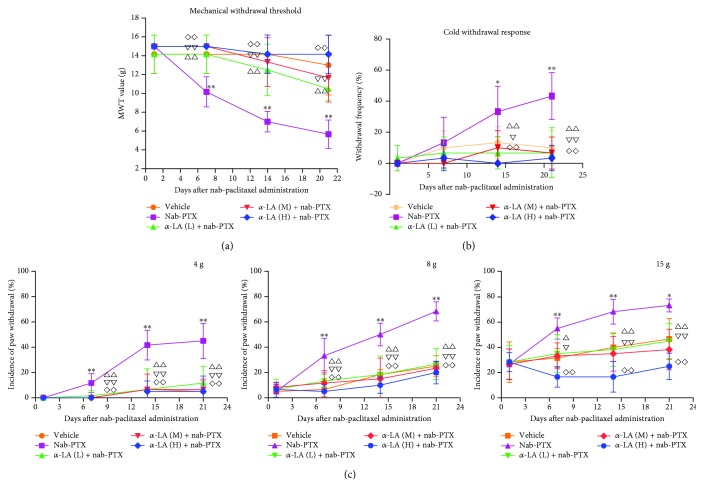
Changes of mechanical and cold withdrawal responses in rats. ^∗^ *p* < 0.05, nab-PTX versus vehicle group; ^∗∗^ *p* < 0.01, nab-PTX versus vehicle group; ^△^ *p* < 0.05, *α*-LA (low dose)+nab-PTX versus nab-PTX group, ^△△^ *p* < 0.01, *α*-LA (low dose)+nab-PTX versus nab-PTX group; ^*∇*^ *p* < 0.05, *α*-LA (middle dose)+nab-PTX versus nab-PTX group; ^*∇∇*^ *p* < 0.01, *α*-LA (middle dose)+nab-PTX versus nab-PTX group; ^◇^ *p* < 0.05, *α*-LA (high dose)+nab-PTX versus nab-PTX group; and ^◇◇^ *p* < 0.01, *α*-LA (high dose)+nab-PTX versus nab-PTX group.

Mechanical and cold allodynia were tested on days 1, 4, 7, 14, and 21 after nab-PTX administration. As shown in Figure 1(a), the bilateral paw withdrawal thresholds of rats in the nab-PTX-treated group decreased significantly following nab-PTX compared with those in the vehicle group (*p* < 0.001), indicating a mirror-like mechanical allodynia. In the 4, 8, and 15 g von Frey tests, nab-PTX-treated rats showed significantly more responses than the controls (Figure 1(c)). The acetone test for cold allodynia also indicated that nab-PTX-treated rats showed significantly more responses than the controls (*p* < 0.001, Figure 1(b)). Furthermore, we investigated whether *α*-LA could ameliorate neuropathic pain and cold allodynia induced by nab-PTX. Our results indicated that all 3 dosage regimens (15, 30, and 60 mg/kg) of *α*-LA had significant effects on neuropathic pain induced by nab-PTX (*p* < 0.05, Figure 1(a)). The mechanical withdrawal thresholds of rats treated with *α*-LA (three dosage regimens) plus nab-PTX increased significantly compared with those of the single nab-PTX-treated ones. In the 4, 8, and 15 g von Frey tests, the rats treated with *α*-LA plus nab-PTX displayed significantly reduced responses than the single nab-PTX-treated group (*p* < 0.05, Figure 1(c)). The cold withdrawal responses of *α*-LA plus nab-PTX-treated rats were also significantly reduced compared with those of the single nab-PTX-treated ones (*p* < 0.05, Figure 1(b)).

### 3.2. Alphalipoic Acid Inhibits Oxidative Stress in Nab-Paclitaxel-Treated Rats

**Figure 2:**
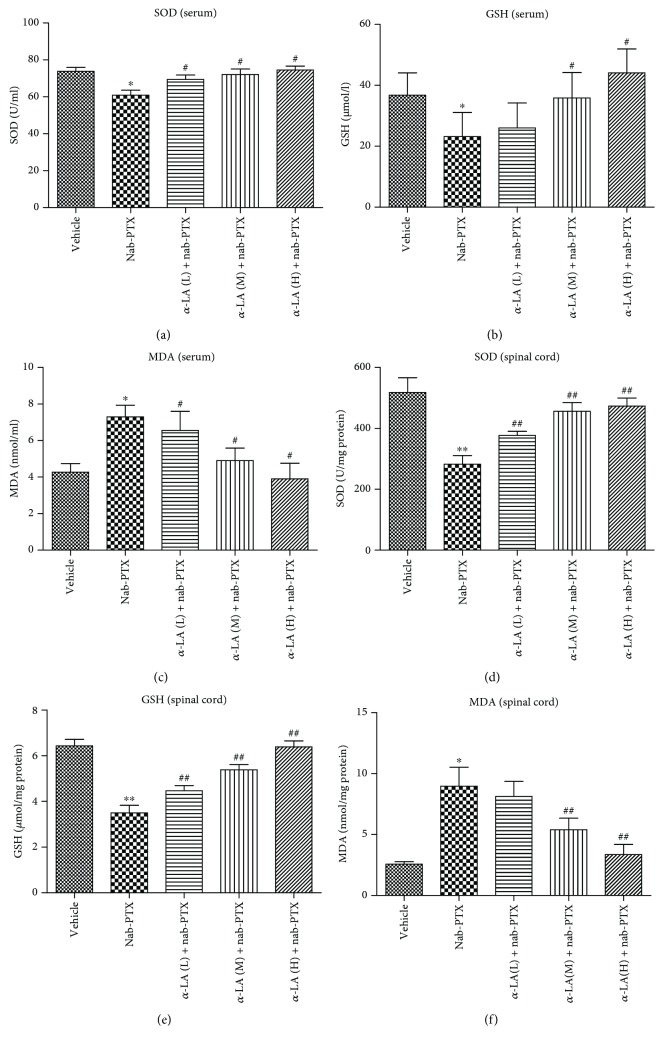
The oxidative activities of the serum and spinal cord tissues in rats. ^∗^ *p* < 0.05 versus vehicle group, ^∗∗^ *p* < 0.01 versus vehicle group, ^#^ *p* < 0.05 versus nab-PTX group, and ^##^ *p* < 0.01 versus nab-PTX group.

The oxidative activities in the serum and spinal cord tissues of rats were measured. The contents of SOD and GSH in both serum and spinal cord tissues were significantly decreased by nab-PTX, suggesting that nab-PTX could disrupt the antioxidant defense systems in the serum and the spinal cord. However, activities of SOD and GSH in both serum and spinal cord tissues could be restored by *α*-LA administration at 3 dosage regimens (15, 30, and 60 mg/kg), respectively (Figures 2(a), 2(b), 2(d), and 2(e)). The contents of MDA in both serum and spinal cord tissues were significantly increased by nab-PTX but significantly decreased by *α*-LA (Figures 2(c) and 2(f)).

### 3.3. Alphalipoic Acid Activates the Nrf2 Pathway in Nab-Paclitaxel-Treated Rats

**Figure 3:**
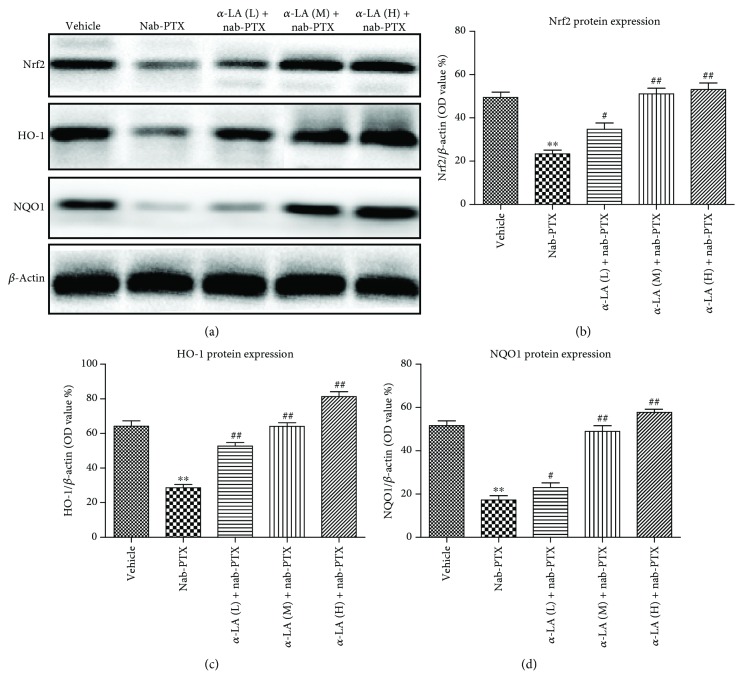
The protein expression of Nrf2, HO-1, and NQO1 of DRGs in rats. ^∗^ *p* < 0.05 versus vehicle group, ^∗∗^ *p* < 0.01 versus vehicle group, ^#^ *p* < 0.05 versus nab-PTX group, and ^##^ *p* < 0.01 versus nab-PTX group.

**Figure 4:**
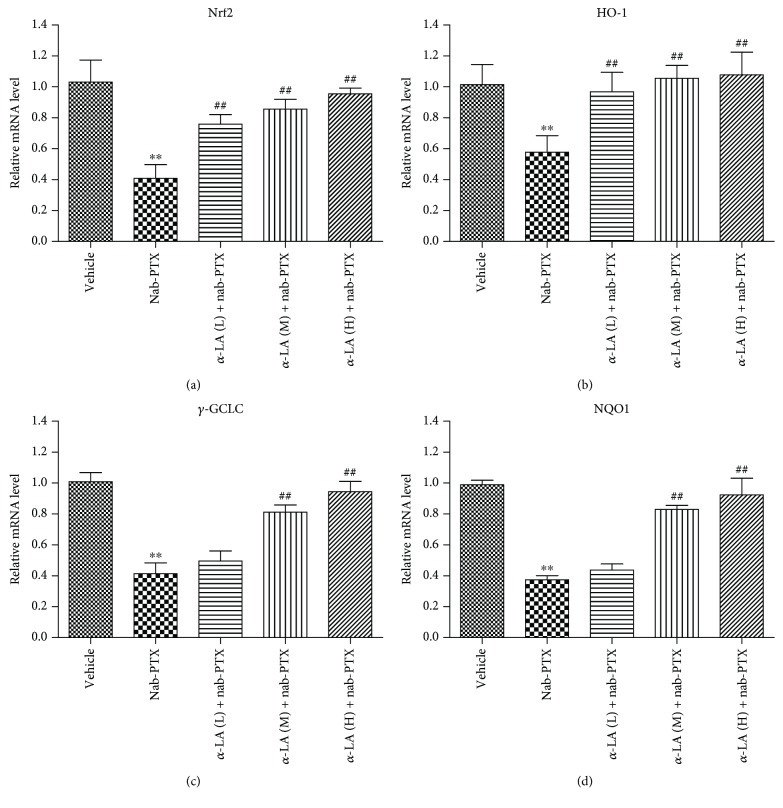
The mRNA levels of Nrf2, HO-1, *γ*-GCLC, and NQO1 of DRGs in rats. (a) Nrf2, (b) HO-1, (c) *γ*-GCLC, and (d) NQO1. ^∗^ *p* < 0.05 versus vehicle group, ^∗∗^ *p* < 0.01 versus vehicle group, ^#^ *p* < 0.05 versus nab-PTX group, and ^##^ *p* < 0.01 versus nab-PTX group.

Compared with the controls, the protein levels of Nrf2, HO-1, and NQO1 in DRGs decreased significantly in the nab-PTX-treated mice. Cotreatment with *α*-LA significantly increased Nrf2, HO-1, and NQO1 protein expressions in the DRGs (Figure 3). The mRNA levels of Nrf2, HO-1, *γ*-GCLC, and NQO1 were significantly decreased by nab-PTX, and *α*-LA administration upregulated the mRNA expressions of these Nrf2 target genes (Figure 4).

### 3.4. Alphalipoic Acid Does Not Reverse the Antitumor Effect of Nab-Paclitaxel in a Mouse Xenograft Model

The administration of *α*-LA (60 mg/kg), nab-PTX (7.4 mg/kg), or vehicle was well tolerated by the mice, without any cases of mortality. The body weight of the mice in the 4 groups had no significant differences (Supplementary Figure 4), indicating that *α*-LA and nab-PTX had no significant impact on the body weight of the mice in our experiment.

**Figure 5:**
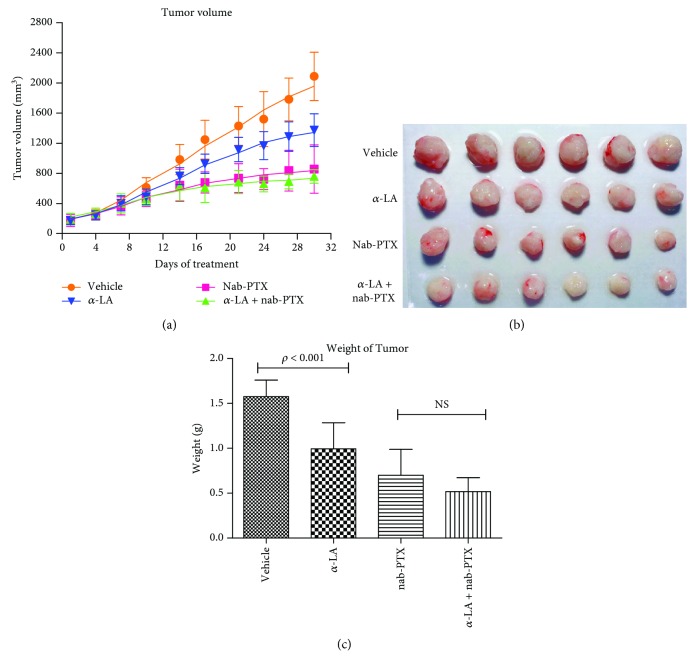
Tumor volume changes and tumor weight of mice.

As shown in Figure 5(a), we found that the growth of CFPAC-1 xenograft tumor was inhibited significantly by *α*-LA as compared with that of controls (*p* < 0.05). However, combinational use of *α*-LA and nab-PTX caused no significant growth inhibition compared with that of single nab-PTX, indicating that *α*-LA had no additional effect on the antitumor effect of nab-PTX. Comparisons of the tumor weight in each group found that the tumor weight of *α*-LA-treated mice was significantly lighter than that of the controls (*p* < 0.001, Figures 5(b) and 5(c)). In addition, although there was no significant difference of tumor weight between *α*-LA plus nab-PTX and single nab-PTX groups, the tumor weight of *α*-LA plus nab-PTX-treated mice tended to be lighter than that of the single nab-PTX-treated ones (Figure 5(c)).

## 4. Discussion

The management of peripheral neuropathy induced by many commonly used chemotherapeutic agents, such as taxanes and platinum drugs, continues to be an important challenge for both clinicians and cancer patients, because it can be extremely painful and/or disabling, causing significant loss of functional abilities and decreasing the quality of life [37]. The current standard care of cancer patients includes the dose reduction and/or discontinuation of chemotherapy treatment which influences chemotherapeutic effect and survival of cancer patients [38].

As a new formulation of PTX, nab-PTX is widely used in clinical practice with a better chemotherapeutic effect and no solvent-related toxicities [15, 39]. However, nab-PTX was reported to have a higher prevalence and severity of peripheral neuropathy in several studies [17, 18]. Our meta-analysis indicated that the incidence of all-grade peripheral neuropathy in cancer patients receiving nab-PTX and solvent-based PTX was 65% (95% CI, 47%-80%) and 54% (95% CI, 44%-63%), respectively. Additionally, the incidence of high-grade peripheral neuropathy between nab-PTX and solvent-based PTX was 16% (95% CI, 11%-23%) and 5% (95% CI, 3%-8%), respectively (*p* < 0.001) (unpublished). Therefore, great attention must be paid to the peripheral neuropathy accompanying the use of nab-PTX in clinical practice. Yet, due to the scarcity of conclusive evidence, no agent is currently recommended for the treatment or prophylaxis of nab-PTX-induced peripheral neuropathy.

Currently, the underlying mechanism of peripheral neuropathy induced by PTX is not entirely understood. Oxidative stress has been known as an imbalance between the free radicals and antioxidant defense system. Neurons were more sensitive to oxidative stress because of the low activity of antioxidant enzymes [40]. Experimental studies supported that there was evidence about PTX-induced neuropathy related to oxidative stress [41]. Alphalipoic acid (*α*-LA) is a physiologic antioxidant that has been examined quite extensively as a treatment for diabetic neuropathy. Moreover, *α*-LA was effective in the treatment of distal sensory motor neuropathy, as well as in the modulation of peripheral neuropathy and pain reduction in diabetic patients [27]. *α*-LA could also ameliorate the docetaxel/cisplatin-induced polyneuropathy [42]. The neuroprotective mechanism of *α*-LA was related to the reduction of oxidative stress from free radical formation [43]. Experimental evidence suggested that *α*-LA could restore the glutathione levels, prevent lipid peroxidation, increase the activity of antioxidant enzymes (such as catalase and superoxide dismutase in peripheral nerves), and increase the blood flow, glucose uptake, and metabolism in peripheral nerves along with nerve conduction velocity (NCV) [44–46]. Moreover, *α*-LA could correct the deficits of neuropeptides (such as substance P and Neuropeptide Y) in the spinal cord [47] and restrain the activation of NF-*κ*B in peripheral nerves [48]. *α*-LA could also exert a neuroprotective action against the reperfusion injury [49], promote the activity of adenosine triphosphate [50], reduce the excess lipid oxidation [45], and ameliorate hyperalgesia [51]. In addition, *α*-LA could protect sensory neurons through its antioxidant and mitochondrial regulatory functions *in vitro* [52]. Although *α*-LA could ameliorate diabetic neuropathy and peripheral neuropathy induced by various types of chemotherapy drugs, it is still unclear whether *α*-LA could have neuroprotective effects on nab-PTX-induced peripheral neuropathy because of the different pathogenic mechanisms [53].

Our study has shown that *α*-LA could significantly ameliorate neuropathic pain and cold allodynia induced by nab-PTX in rats via Nrf2 activation and oxidative stress inhibition, suggesting that *α*-LA ameliorated the peripheral neuropathy in this experimental model by protecting against oxidative system damage caused by nab-PTX. Although our experimental number of 6 per group sounds small to permit consistent conclusions for behavioral assessments, the results of power analysis indicate that it is acceptable in the present study (data not shown). After determining the neuroprotective effects of *α*-LA, we were not sure whether *α*-LA could influence the chemotherapeutic effect of nab-PTX. Therefore, we established a subcutaneous xenograft model of pancreatic cancer and found that *α*-LA had a potential antitumor effect, although the antitumor effect was not significant when combined with nab-PTX. Several studies also indicated that *α*-LA could sensitize lung cancer cells to chemotherapeutic agents [54], promote synergistic antitumor effects [55], and inhibit breast cancer cell proliferation [56].

Although our study has confirmed that *α*-LA could ameliorate the peripheral neuropathy induced by nab-PTX without diminishing the chemotherapeutic effect, there are still some limitations in our study. First, our study was done in rats and whether this conclusion is also applicable to humans would require further study. Additionally, we established a subcutaneous xenograft model of pancreatic cancer; whether the results could also be consistent in the orthotopic xenograft tumor model in consideration of the complexity of pancreatic cancer, further research needs to be performed.

## 5. Conclusions

In conclusion, the data presented here suggests that *α*-LA could significantly prevent oxidative stress and peripheral neuropathy in nab-PTX-treated rats through the Nrf2 signalling pathway without diminishing the chemotherapeutic effect of nab-PTX in a subcutaneous xenograft tumor model. To employ this inexpensive, relatively safe neuroprotective drug in chemotherapeutic regimens that induce neuropathy, larger preclinical and clinical studies need to be performed.

## Figures and Tables

**Table 1:** Sequences of the primers used for Nrf2, HO-1, *γ*-GCLC, NQO1, and GAPDH.

Gene	Sequences of primers
Nrf2	Forward: 5′-TTGGCAGAGACATTCCCATTTGTA-3′
	Reverse: 5′-GAGCTATCGAGTGACTGAGCCTGA-3′
HO-1	Forward: 5′-AGGTGCACATCCGTGCAGAG-3′
	Reverse: 5′-CTTCCAGGGCCGTATAGATATGGTA-3′
*γ*-GCLC	Forward: 5′-CTGCACATCTACCACGCAGTCA-3′
	Reverse: 5′-ATCGCCGCCATTCAGTAACAA-3′
NQO1	Forward: 5′-TGGAAGCTGCAGACCTGGTG-3′
	Reverse: 5′-CCCTTGTCATACATGGTGGCATAC-3′
GAPDH	Forward: 5′-GGCACAGTCAAGGCTGAGAATG-3′
	Reverse: 5′-ATGGTGGTGAAGACGCCAGTA-3′

## Data Availability

All the data used to support the findings of this study are available from the corresponding author upon request.

## Supplementary Materials

Supplementary Materials Figure S1: experimental groups and protocol of study on neurotoxicity protection by *α*-LA. Figure S2: experimental groups and protocol of study on antitumor effect by *α*-LA. Figure S3: changes of body weight (g) in rats. Figure S4: changes of body weight (g) in nude mice. Supplementary File 1: Experimental Animals Ethics Committee of School of Pharmacy Fudan University.
