# Supplementary material for: Alphalipoic Acid Prevents Oxidative Stress and Peripheral Neuropathy in Nab-Paclitaxel-Treated Rats through the Nrf2 Signalling Pathway
Source: Oxid Med Cell Longev. 2019 Feb 10;2019:3142732. doi: 10.1155/2019/3142732 (PMC6387730; doi:10.1155/2019/3142732)
Supplement: Supplementary Materials — Figure S1: experimental groups and protocol of study on neurotoxicity protection by α-LA. Figure S2: experimental groups and protocol of study on antitumor effect by α-LA. Figure S3: changes of body weight (g) in rats. Figure S4: changes of body weight (g) in nude mice. Supplementary File 1: Experimental Animals Ethics Committee of School of Pharmacy Fudan University. [file 3142732.f1.pdf]

Supplementary Materials

1. Supplementary Figure 1: Experimental groups and protocol of study on neurotoxicity protection by α-LA

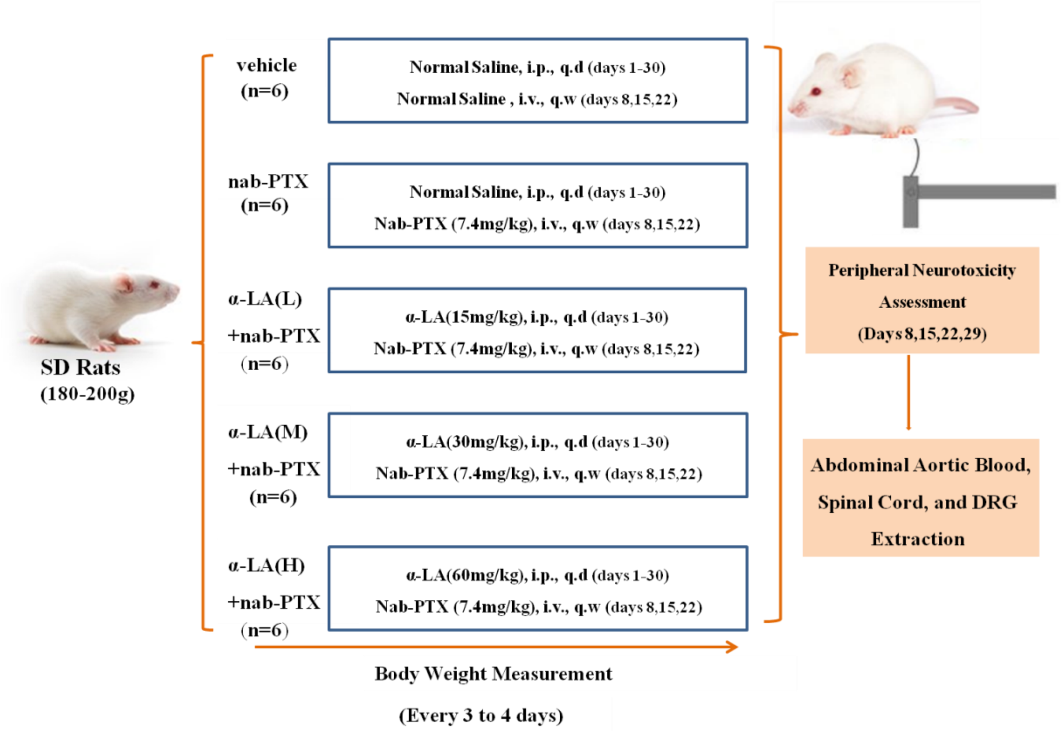

2. Supplementary Figure 2: Experimental groups and protocol of study on anti-tumor effect by α-LA

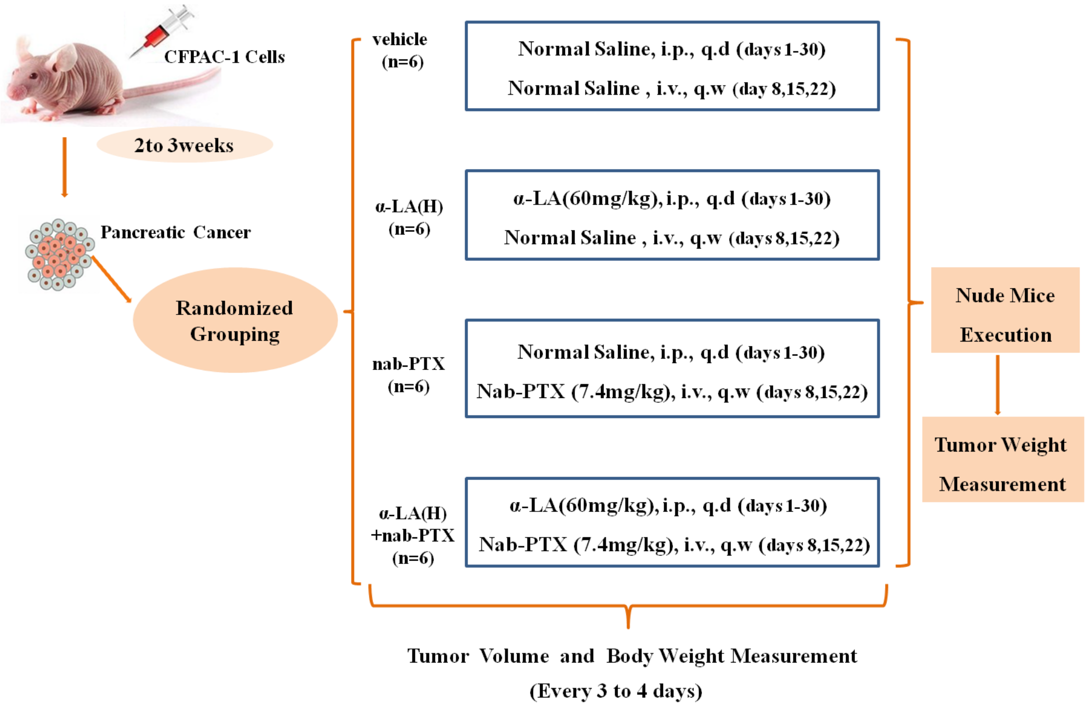

3. Supplementary Figure 3: Changes of body weight (g) in rats

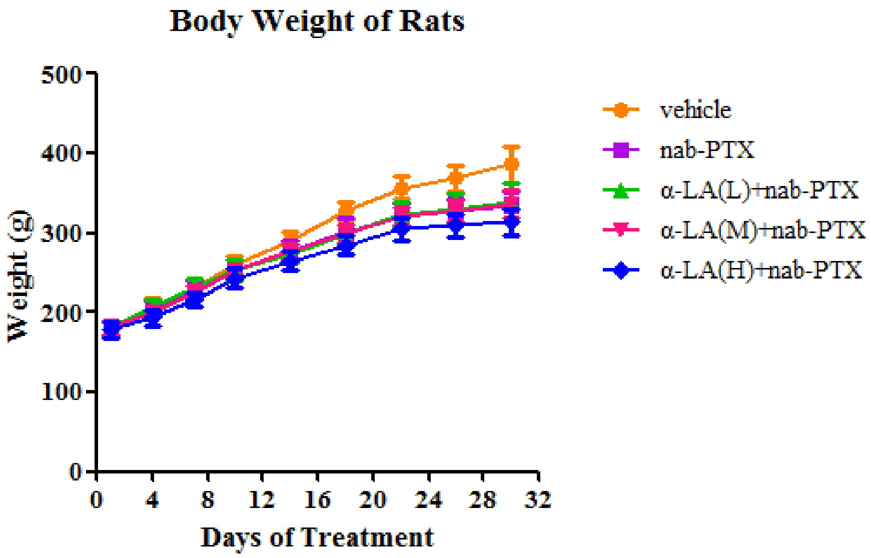

4. Supplementary Figure 4: Changes of body weight (g) in nude mice

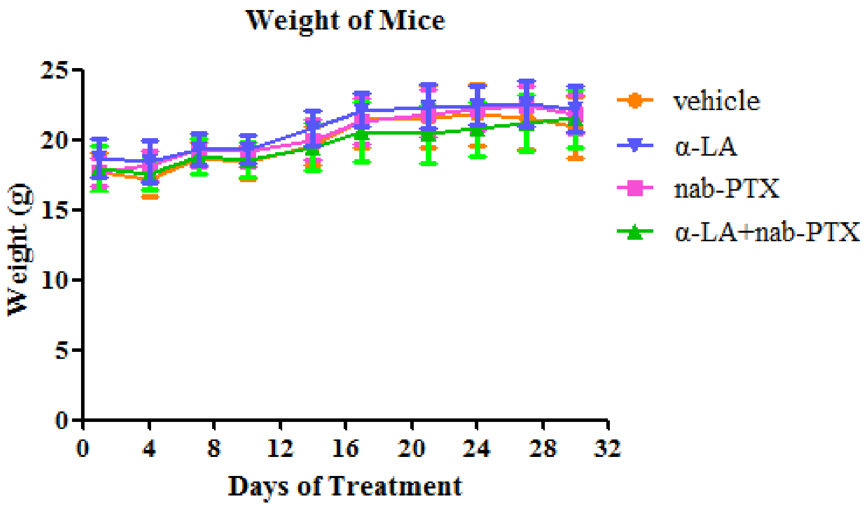

5. Supplementary File 1: Experimental Animals Ethics Committee of School of Pharmacy Fudan University

Experimental Animal Ethics Committee of School of Pharmacy Fudan University

Description outlining the justification for using animals, the proposed use of animals and the significance of the research documentation

Ethical approval number: (2017-10-LY-CWM-01)

**Project title**	Efficacy of α-lipoic acid on peripheral neuropathy and anti-tumor effect of nab-paclitaxel				
**Category**	**Foundation**______ **Clinical trials**______ **Medication**__√__				
**Project source**	Self-initiated project				
**Research institute**	School of Pharmacy Fudan University			**Head**	Mingwei Wang
**Department/ Faculty**	Clinical Pharmacy	**Principal investigator**	Weimin Cai	**Title**	Professor

Ethics review comments

Δ Agree	Δ Agree after modification	Δ Disagree (Termination or suspension of the project)
√		

Review comments

The project is compliance with ethical standards and is approved through the examination and discussion of the materials submitted.

Chairman (**signature**) Cheng Nengneng

Experimental Animal Ethics Committee

School of Pharmacy, Fudan University

(Stamp)

Date: Oct. 10, 2017
